# Characterizing the Role of Brain Derived Neurotrophic Factor Genetic Variation in Alzheimer’s Disease Neurodegeneration

**DOI:** 10.1371/journal.pone.0076001

**Published:** 2013-09-26

**Authors:** Robyn A. Honea, Carlos Cruchaga, Rodrigo D. Perea, Andrew J. Saykin, Jeffrey M. Burns, Daniel R. Weinberger, Alison M. Goate, For the Alzheimer’s Disease Neuroimaging Initiative (ADNI)

**Affiliations:** 1 Department of Neurology, University of Kansas Alzheimer’s Disease Center, University of Kansas Medical Center, Kansas City, Kansas, United States of America; 2 Department of Psychiatry & Neurology, Washington University School of Medicine, St. Louis, Missouri, United States of America; 3 Center for Neuroimaging, Division of Imaging Sciences, Department of Radiology, Center for Computational Biology and Bioinformatics, Indiana University School of Medicine, Indianapolis, Indiana, United States of America; 4 Department of Medical and Molecular Genetics, Indiana University School of Medicine, Indianapolis, Indiana, United States of America; 5 Lieber Institute for Brain Development, Johns Hopkins Medical Campus, Baltimore, Maryland, United States of America; 6 Departments of Psychiatry, Neurology, Neuroscience and the McKusick-Nathans Institute of Genetic Medicine, Johns Hopkins School of Medicine, Baltimore, Maryland, United States of America; Nathan Kline Institute and New York University School of Medicine, United States of America

## Abstract

There is accumulating evidence that neurotrophins, like brain-derived neurotrophic factor (*BDNF*), may impact aging and Alzheimer’s Disease. However, traditional genetic association studies have not found a clear relationship between *BDNF* and AD. Our goal was to test whether *BDNF* single nucleotide polymorphisms (SNPs) impact Alzheimer’s Disease-related brain imaging and cognitive markers of disease. We completed an imaging genetics study on 645 Alzheimer’s Disease Neuroimaging Initiative participants (ND=175, MCI=316, AD=154) who had cognitive, brain imaging, and genetics data at baseline and a subset of those with brain imaging data at two years. Samples were genotyped using the Illumina Human610-Quad BeadChip. 13 SNPs in BDNF were identified in the dataset following quality control measures (rs6265(Val66Met), rs12273363, rs11030094, rs925946, rs1050187, rs2203877, rs11030104, rs11030108, rs10835211, rs7934165, rs908867, rs1491850, rs1157459). We analyzed a subgroup of 8 SNPs that were in low linkage disequilibrium with each other. Automated brain morphometric measures were available through ADNI investigators, and we analyzed baseline cognitive scores, hippocampal and whole brain volumes, and rates of hippocampal and whole brain atrophy and rates of change in the ADAS-Cog over one and two years. Three out of eight *BDNF* SNPs analyzed were significantly associated with measures of cognitive decline (rs1157659, rs11030094, rs11030108). No SNPs were significantly associated with baseline brain volume measures, however six SNPs were significantly associated with hippocampal and/or whole brain atrophy over two years (rs908867, rs11030094, rs6265, rs10501087, rs1157659, rs1491850). We also found an interaction between the *BDNF* Val66Met SNP and age with whole brain volume. Our imaging-genetics analysis in a large dataset suggests that while BDNF genetic variation is not specifically associated with a diagnosis of AD, it appears to play a role in AD-related brain neurodegeneration.

## Introduction

Alzheimer’s Disease (AD) is a neurodegenerative disorder that results in the increased production of amyloid-B peptide and Tau protein hyperphosphorylation, as well as the degeneration and death of neurons. Although the causes of late-onset AD pathology are unknown, family studies have demonstrated that complex genetic and environmental mechanisms contribute to disease risk [1,2]. Since the elucidation of the APOE locus in 1993 [3], over 660 candidate genes for AD risk have been identified, however results are inconsistent between studies [4]. To further characterize complex genes associated with AD, a growing number of studies are using an intermediate phenotype approach, which utilizes biomarkers, such as structural brain imaging of hippocampal atrophy, as endpoints in genetic analyses of risk.

The brain-derived neurotrophic factor (*BDNF*) gene has been a candidate risk gene for diseases involving memory loss due to its facilitation of long-term plasticity in the hippocampus, a function that breaks down during the onset of AD. Moreoever, accumulating evidence points to a protective role for *BDNF* in neurons through increased neuroprotection [5,6], and reduction of Aβ peptide [7]. Post-mortem studies show that *BDNF* expression is severely decreased in the hippocampus, temporal, and frontal cortex in AD [8,9]. Thus, decreased *BDNF* in the brain might contribute to advanced aging as well as AD [10]. There is a well-known functional single nucleotide polymorphism (SNP) in the 5’ proregion of the human *BDNF* gene at nucleotide 196. The SNP results in a Valine (Val) to Methionine (Met) amino acid substitution at codon 66 (Val66Met, rs6265, G>A). When Val-BDNF and Met-BDNF are produced together in neuronal cells they form heterodimers, which alter *BDNF* trafficking and decrease secretion of *BDNF* [11]. Imaging genetics studies, which may be more sensitive then traditional gene-association studies, have recently identified a role for the *BDNF* Val66Met SNP in hippocampal volume loss [12], memory impairments [13], reduced medial temporal lobe activity [14] and modified experience-dependent plasticity in the motor cortex [15] in healthy humans. Increasing age may also mediate the effects of the Val66Met SNP [16]. Some studies have also shown that variation in this Val66Met polymorphism may increase risk for Alzheimer’s Disease and impact cognitive performance [17,18]. However, there is still conflicting evidence of the relationship between *BDNF* genetic variation and AD [19-22], with several studies showing no relationship. Finally, other functional SNPs in *BDNF* have been identified that may impact human brain function [23], demonstrating the importance of investigating multiple *BDNF* SNPs using an AD phenotype approach to clarify *BDNF*’s role in brain neurodegeneration.

Thus, our goal was to use neuroimaging and cognitive phenotypes that have been associated with AD, and test whether genetic variation in *BDNF* impacts these phenotypes in a large sample from the Alzheimer’s Disease Neuroimaging Initiative (ADNI). ADNI is an NIH-sponsored, multi-site study assessing MRI, biological, clinical and neuropsychological traits to measure the progression of mild cognitive impairment (MCI) and early AD. This large dataset includes approximately 800 participants with imaging data, cognitive, and genetic data at several time points. There has been one analysis of *BDNF* Val66Met and brain metabolism in the ADNI sample [24], however no studies have investigated the relationship of several *BDNF* SNPs to AD endophenotypes in this dataset to date.

## Materials and Methods

### Subjects

Data used in the preparation of this article were obtained from the Alzheimer’s Disease Neuroimaging Initiative (ADNI) database (adni.loni.ucla.edu). The ADNI was launched in 2003 by the National Institute on Aging (NIA), the National Institute of Biomedical Imaging and Bioengineering (NIBIB), the Food and Drug Administration (FDA), private pharmaceutical companies and non-profit organizations, as a $60 million, 5-year public-private partnership. The primary goal of ADNI has been to test whether serial magnetic resonance imaging (MRI), positron emission tomography (PET), other biological markers, and clinical and neuropsychological assessment can be combined to measure the progression of mild cognitive impairment (MCI) and early Alzheimer’s disease (AD). Determination of sensitive and specific markers of very early AD progression is intended to aid researchers and clinicians to develop new treatments and monitor their effectiveness, as well as lessen the time and cost of clinical trials.

The Principal Investigator of this initiative is Michael W. Weiner, MD, VA Medical Center and University of California – San Francisco. ADNI is the result of efforts of many co-investigators from a broad range of academic institutions and private corporations, and subjects have been recruited from over 50 sites across the U.S. and Canada. The initial goal of ADNI was to recruit 800 subjects but ADNI has been followed by ADNI-GO and ADNI-2. To date these three protocols have recruited over 1500 adults, ages 55 to 90, to participate in the research, consisting of cognitively normal older individuals, people with early or late MCI, and people with early AD. The follow up duration of each group is specified in the protocols for ADNI-1, ADNI-2 and ADNI-GO. Subjects originally recruited for ADNI-1 and ADNI-GO had the option to be followed in ADNI-2. For up-to-date information, see www.adni-info.org. Data for the present analysis were downloaded from the ADNI web site (ADNI-1 data) in November, 2010.

The study reported here involved 745 subjects who had MRI scans at least at baseline, and some at 24 months, as well as genetic and cognitive data. Of those subjects, 48 subjects were excluded for technical reasons, such as major hardware upgrades during the study (at two sites), miscalibration of image resolution, excess movement, or failure of one or more automatic processing methods. Fifty-two subjects were excluded because they did not meet the genetics quality control (see below for criteria). The final dataset for analysis included, 175 normal, 316 mild cognitive impairment, and 154 Alzheimer’s disease subjects. The main demographic and clinical data, including apolipoprotein E4 (ApoE4) carrier data, are summarized in Table **1**.

**Table 1:** Baseline Demographic, Clinical, and Neuroimaging Characteristics of Study Participants.

	**Means (SD**)** where given**		
	**Controls**	**MCI**	**AD**
**Characteristic**	**n=175**	**n=316**	**n=154**
**Age, y**	76.1 (4.9)	75.4 (7.2)	75.4 (7.6)
**Male sex, No. (%)**	96 (54.8)	204 (64.5)	82 (53.2)
**Education level, y**	16.2 (2.7)	15.8 (2.9)	14.9 (2.9)
**APOE ε4, minor allele No. (%)**	55 (31.4)	172 (54.4)	90 (58.4)
**GDS score**	1.0 (1.2)	1.54 (1.4)	1.6 (1.4)
**Clinical Dementia Rating Global Score**	.00 (.0)	.49 (.03)	.72 (.23)
**MMSE**	29.1 (.95)	27.1 (1.8)	23.5 (2.0)
**ADAS-COG score**	6.05 (2.7)	11.4 (4.4)	18.2 (5.9)
**ADAS-COG score 1 year change**⌃	-.434 (3.0)	1.04 (4.4)	4.06 (6.4)
**ADAS-COG score 2 year change** ω	-.190 (2.9)	2.94 (5.9)	9.27 (9.1)
**Normalized Whole Brain Volume**°	.685 (.02)	.671 (.03)	.660 (.03)
**Normalized Left Hippocampal Volume**°	.242 (.03)	.212 (.03)	.198 (.03)
**Normalized Right Hippocampal Volume**°	.254 (.02)	.224 (.03)	.210 (.03)
Percent Whole Brain Atrophy (2 years)*	-.925 (.95)	-1.62 (1.3)	-2.71 (1.5)
Percent Left Hippocampal Atrophy (2 years)*	-1.82 (1.7)	-4.14 (3.3)	-6.56 (3.1)
Percent Right Hippocampal Atrophy (2 years)*	-1.61 (1.9)	-4.23 (3.8)	-7.03 (3.9)

MCI, Mild Cognitive Impairment; AD, Alzheimer’s disease; n, number; y, years; GDS, Geriatric Depression Scale Total score; MMSE, Mini-Mental Status Exam total score; ADAS-COG, Alzheimer’s disease assessment scale- cognitive subscale, Total 11; Normalized= normalized to total intracranial volume. For ADAS-COG change scores, an increased score represents cognitive decline, as higher scores equal worse performance. ⌃For 1-year change in ADAS-COG Total 11 score, the sample was: ND = 164, MCI = 286, AD = 132. ω For 2-year change in ADAS-COG Total 11 score, the sample was: ND = 159, MCI = 245, AD = 110. ° For baseline imaging measures, we used a subsample of individuals with baseline imaging data (Controls = 166, MCI = 281, AD = 131). * For percent atrophy measures, we used a subsample of individuals that had both baseline and 24 month brain images (Controls = 127, MCI = 179, AD = 75).

### Ethics Statement

Ethics approval was obtained for each institution involved. This study was conducted according to Good Clinical Practice guidelines, the Declaration of Helsinki, US 21CFR Part 50- Protection of Human Subjects, and Part 56- Institutional Review Boards, and pursuant to state and federal HIPAA regulations. Written informed consent for the study was obtained from all subjects and/or authorized representatives and study partners before protocol-specific procedures are carried out. Institutional Review Boards were constituted according to applicable State and Federal requirements for each participating location. The protocols were submitted to appropriate Boards and their written unconditional approval obtained and submitted to Regulatory Affairs at the Alzheimer’s Disease Neuroimaging Initiative Coordinating Center (ADNI-CC) prior to commencement of the study. Further information about ADNI can be obtained from www.adni-info.org.

### Genetics Data

Samples were genotyped using the Illumina Human610-Quad BeadChip. All samples and genotypes underwent stringent quality control (QC). Genotype data were cleaned by applying minimum call rates (98%) and minimum minor allele frequencies (0.02). SNPs not in Hardy-Weinberg equilibrium (P< 1x 10^−6^) were excluded. We tested for unanticipated duplicates and cryptic relatedness using pairwise genome-wide estimates of proportion identity-by-descent. When a pair of identical samples or a pair of samples with cryptic relatedness was identified, the sample with a higher number of SNPs that passed QC were prioritized. Eigenstrat [25] was used to calculate principal component factors for each sample and confirm the ethnicity of the samples. Thirteen SNPs in *BDNF* passed QC (rs6265(Val66Met), rs12273363, rs11030094, rs925946, rs1050187, rs2203877, rs11030104, rs11030108, rs10835211, rs7934165, rs908867, rs1491850, rs1157459). We used the SNP Annotation and Proxy Search (SNAP) [26] to determine proxy based linkage disequilibrium using HapMap, and detailed other information from the selected SNPs from the International Hapmap Project (http://hapmap.ncbi.nlm.nih.gov/). We tested for an association of *BDNF* SNP allele frequency to Alzheimer’s Disease in the nondemented and AD groups using Pearson’s [chi]^2^ test (Table **2**). We did not include the MCI group in this test due to heterogeneity of the sample. We also list SNP position, location, and type in Table **2**. The linkage disequilibrium (LD) between genotyped variants can be found in Table **S1**. For our AD phenotype analysis we analyzed a subgroup of 8 SNPs, 7 which were independent of each other (r^2^ <.4) (rs11030108, rs10501087, rs908867, rs11030094, rs1491850, rs1157679, rs12273363) as well as rs6265 (Val66Met), which is in LD with rs10501087 (r^2^ = .817), however, we wanted to include it for comparability with the literature.

**Table 2:** BDNF SNP Location and Association Details.

**SNP**	**Major/ Minor**	**Chromosome Position^a^**	**Intermarker Distance^b^**	**Location**	**HapMap CEU MAF**	**Control**	**AD**	**χ^2^**	**p value**
rs11030094	A/G	27659775	0	Intergenic	0.351	AA: 29.7% (52)	AA: 29.2% (45)	1.62	0.805
						AG: 49.1% (86)	AG: 48.1% (74)		
						GG: 21.1% (37)	GG: 22.7% (35)		
rs925946	G/T	27667202	7427	Intergenic	0.358	GG: 47.4% (83)	GG: 46.1% (71)	4.395	0.355
						GT: 45.7% (80)	GT: 40.9% (63)		
						TT: 6.9% (12)	TT: 13% (20)		
rs10501087	T/C	27670108	2906	Intergenic	0.23	TT: 62.9% (110)	TT:69.5% (107)	2.331	0.32
						CC/CT: 37.1% (65)	CC/CT: 30.5% (47)		
rs2203877	T/C	27670910	802	Intergenic	0.434	TT: 52.6% (92)	TT: 51.3% (79)	0.951	0.622
						CC/CT: 47.4% (83)	CC/CT: 48.7% (75)		
rs6265	G/A	27679916	9006	Nonsynonymous	0.175	GG: 66.9% (117)	GG: 70.8% (109)	1.313	0.428
						AA/AG: 33.1% (58)	AA/AG: 29.2% (45)		
rs11030104	A/G	27684517	4601	Intron	0.2	AA: 63.4% (111)	AA: 69.5% (107)	2.563	0.278
						GG/AG: 36.6% (64)	GG/AG: 30.5% (47)		
rs11030108	G/A	27695464	10947	Intron	0.367	GG: 47.4% (83)	GG: 44.8% (69)	0.751	0.687
						AA/AG: 52.6% (92)	AA/AG: 55.2% (85)		
rs10835211	G/A	27701365	5901	Intron	0.3	GG: 57.1% (100)	GG: 53.2% (82)	0.535	0.765
						AA/AG: 42.9% (75)	AA/AG: 46.8% (72)		
rs7934165	G/A	27731983	30618	Intron	0.425	GG: 25.7% (45)	GG:25.3% (39)	1.797	0.773
						AG: 49.1% (86)	AG: 46.8% (72)		
						AA: 25.1% (44)	AA: 27.9% (43)		
rs1157659	T/C	27741419	9436	Intergenic	0.44	TT: 29.1% (51)	TT: 23.4% (36)	0.496	0.341
						CT: 50.3% (88)	CT: 54.5% (84)		
						CC: 20.6% (36)	CC: 22.1% (34)		
rs12273363	T/C	27744859	3440	Upstream	0.19	TT: 62.3% (109)	TT:60.4% (93)	0.405	0.725
						CC/CT: 37.7% (66)	CC/CT: 39.6% (61)		
rs908867	C/T	27745764	905	Upstream	0.117	CC: 85.1% (149)	CC: 82.5% (127)	0.305	0.512
						TT/CT: 14.9% (26)	TT/CT: 17.5% (27)		
rs1491850	T/C	27749725	3961	Intergenic	0.442	TT: 32.0% (56)	TT: 34.4% (53)	0.87	0.6
						CT: 47.4% (83)	CT: 46.8% (72)		
						CC: 20.6% (36)	CC: 18.8% (29)		

MAF- Minor allele frequency. ^a^ Chromosome 11 position according to NCBI Build 37.1 genome assembly, ^b^ In base pairs. In cases where the minor allele frequency was <.10, heterozygous and minor-allele homozygous subgroups were grouped together.

### ADNI Measures

We used hippocampal and whole brain volume (WBV) data from the Anders Dale Lab (UCSD) available as part of the ADNI secondary imaging data downloads. Details on their neuroimaging processing methods are published elsewhere [27]. For normalization calculations we divided by the UCSD estimated intracranial volumes. Normalized left hippocampus, right hippocampus, and whole brain volumes were used as the baseline brain imaging endophenotypes for genetics analysis. There were 166 healthy controls, 281 MCI, and 131 AD individuals with secondary imaging volumes from UCSD that passed quality controls, were available for download, and had corresponding genetics data. We calculated a percent change of normalized left and right hippocampal volume, and WBV, using normalized baseline and 24 month imaging data. For percent atrophy measures, we used a subsample of data that had brain imaging measures at baseline and 2 years that passed UCSD quality controls (Controls = 127, MCI = 179, AD = 75). As a marker for disease-related cognitive change, we used the Alzheimer’s Disease Assessment Scale- cognitive subscale (ADAS-Cog) total score at baseline and calculated 1 and 2-year change scores (subtracting 1 and 2 year scores from baseline) for longitudinal data. Because both the ADAS-Cog and the hippocampal and whole brain imaging measures were not normally distributed across our sample, we log-transformed all measures and did statistics on these log-transformed measures.

### Neuroimaging Statistics

We completed statistics across all diagnostic groups (controlling for disease severity, age, sex, and ApoE genotype), and within diagnostic groups separately (controlling for age, sex, and ApoE genotype). For tests of association of SNP with disease phenotype we used univariate statistics within the general linear model, controlling for multiple test corrections using Bonferroni correction. In cases where the minor allele frequency was <.10, heterozygous and minor-allele homozygous subgroups were grouped together, with means and statistics representing the joint group. Because *BDNF* rs6265 has been shown to have differing effects across age and between sexes, we also tested for gene-by-age and gene-by-sex interactions on imaging and cognitive endophenotypes with this particular SNP, both within and across diagnosis groups.

## Results

None of the tested allele frequencies of the SNPs were associated with a diagnosis of Alzheimer’s Disease (Table **2**). Overall, three SNPs were significantly associated with the ADAS-Cog score at baseline or change in ADAS-Cog over time, which is a measure of disease severity (rs1157659, rs11030094, rs11030108) (Table **3**). Of these, rs1157659 was significantly associated with baseline ADAS-Cog score in the overall group, as well as the MCI group separately. In regards to a relationship of *BDNF* with cognitive decline, there was a significant association with rs11030094 and change in ADAS-Cog score in the ND group, and rs11030108 in the MCI group. There were no significant relationships between *BDNF* genotype and cognitive measures in the AD group.

**Table 3:** Significant results from analysis of imaging and cognitive phenotypes with BDNF SNPs.

			**Means (SD**)** of raw variables**			
**Group**		**SNP**	**Major Allele HZ**	**Heterozygous**	**Minor Allele HZ**	**P Value**
**All Diagnoses**	**Log-transformed Variable**					
**Cognitive**	**Baseline ADAS**	rs1157659	10.73 (5.9)	12.14 (6.4)	11.2 (5.9)	**0.025**
**Imaging**	**2-Year R Hippo Atrophy**	rs908867	-3.82 (3.8)	-4.37 (4.2)		**0.01**
**ND**						
**Cognitive**	**1-Year ADAS Δ**	rs11030094	.864 (3.37)	-.66 (3.22)	1.61 (4.8)	**0.021**
**Imaging**	**Baseline R Hippo**	rs11030094	.261 (.03)	.251 (.03)	.252 (.03)	0.056
	**2-Year WBV Atrophy**	rs11030094	-1.19 (.80)	-.92 (.88)	-.58 (1.19)	**0.048**
	**2-Year R Hippo Atrophy**	rs6265	-1.38 (1.8)	-2.05 (2.2)		**0.027**
		rs10501087	-1.41 (1.8)	-1.96 (1.9)		**0.048**
	**2-Year L Hippo Atrophy**	rs1157659	-1.33 (1.6)	-2.25 (1.8)	-1.52 (1.1)	**0.025**
**MCI**						
**Cognitive**	**Baseline ADAS**	rs1157659	10.4 (3.8)	12.1 (4.5)	11.1 (4.2)	**0.012**
	**1-Year ADAS Δ**	rs11030108	.441 (4.5)	1.65 (4.3)		**0.028**
**AD**						
**Imaging**	**2-Year R Hippo Atrophy**	rs908867	-6.76 (3.5)	-8.51 (5.3)		**0.025**
	**2-Year L Hippo Atrophy**	rs1491850	-6.66 (3.3)	-5.96 (2.5)	-8.25 (3.9)	**0.048**

Table 3 presents significant results from univariate analysis of variance of log-transformed AD phenotypes and BDNF SNPs, first across all diagnoses groups, then split into separate analyses. In cases where the minor allele frequency was <.10, heterozygous and minor-allele homozygous subgroups were grouped together, with means and statistics representing the joint group. P-values are corrected for multiple comparisons. HZ = homozygotes, SD= standard deviation, L= Left, R= Right, Hippo = normalized hippocampal volume, ADAS = ADAS Total 11 Cognitive Score, 1-Year Δ = 1-Year change score, 2-Year Δ = 2-Year change score, WBV= normalized whole brain volume, ND = Nondemented, MCI = Mild Cognitive Impairment, AD = Alzheimer’s Disease. Atrophy measures are annualized percent change per year. Statistics from All-Diagnoses included age, sex, APOE genotype, and diagnostic classification as covariates. Statistics from the ND, MCI, and AD analyses included age, sex, and APOE genotype as covariates.

No SNPs were significantly associated with baseline brain volume measures, however there was a trend for a relationship between baseline right hippocampal volume and rs11030094 in the ND group (p = .056). Six SNPs were significantly associated with hippocampal and/or whole brain atrophy over two years (rs908867, rs11030094, rs6265, rs10501087, rs1157659, rs1491850). Only rs11030094 was associated with percent change in whole brain volume (p = .048), and this was in the ND group alone. In the combined analysis only rs908867 was associated with an imaging endophenotype, percent of right hippocampal atrophy over two years (p = .010). ND subjects had the highest number of associations with imaging endophenotypes, genetic variation in rs6265 (p = .027) and rs10501087 (p = .048) (in LD with each other) was associated with percent of right hippocampal atrophy over two years, and variation in rs1157659 was associated with left hippocampal atrophy (p = .025). There were no significant associations between *BDNF* genotype and imaging variables in the MCI group. In the AD group, variation in rs908867 was associated with percent of right hippocampal atrophy (p = .025), and variation in rs1491850 was associated with percent left hippocampal atrophy over 2 years (p = .048). Significant results are detailed in Table **3**.

**Figure 1:**
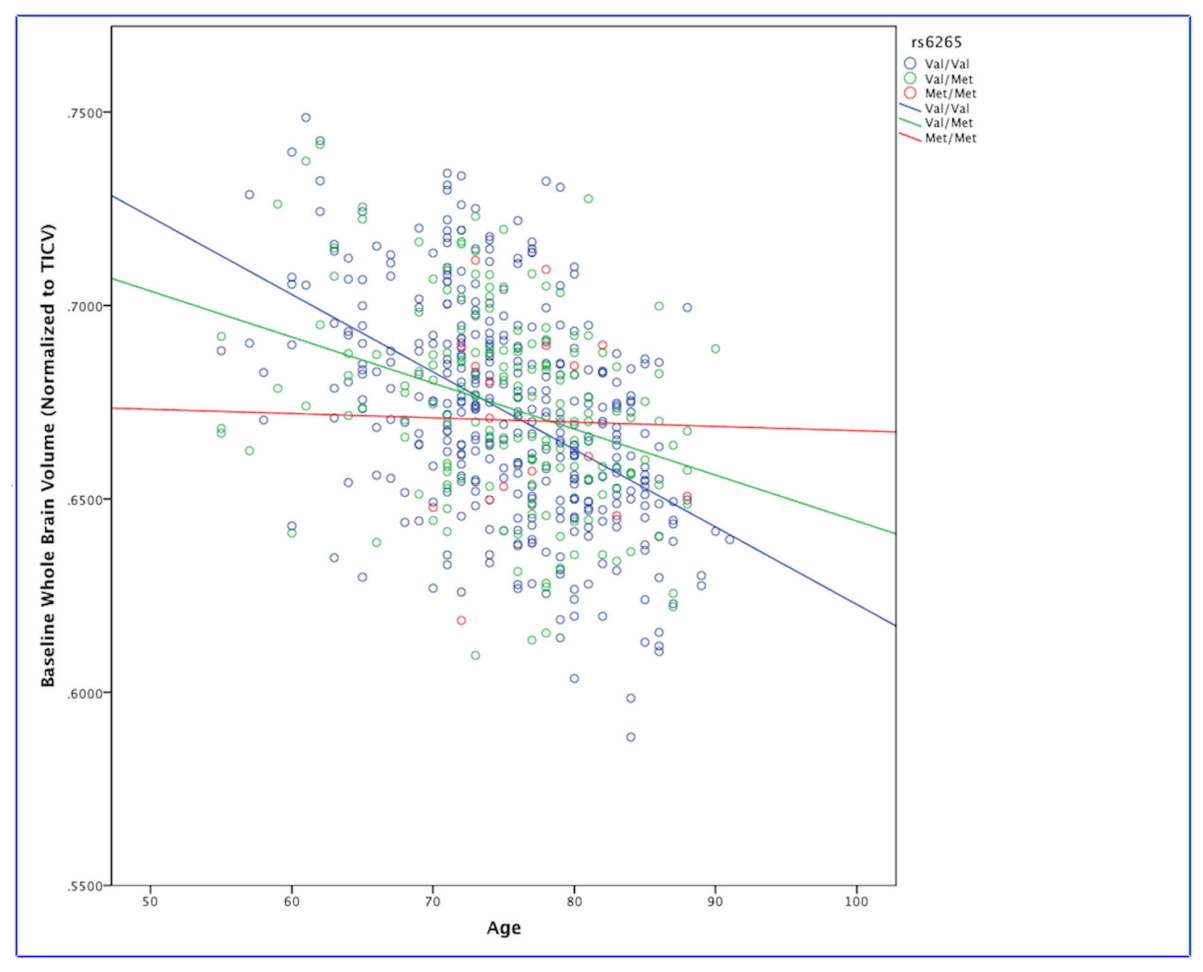
Interaction between the BDNF Val66Met (rs6265) variant with age on whole brain volume.

In our analysis of gene by age interactions with the rs6265 SNP we found a significant interaction (p<.005) with baseline whole brain volume measure (controlling for sex, diagnostic classification, and APOE) in the whole dataset, such that Val/Val homozygotes (n=387) had lower whole brain volume with increasing age compared to Val/Met (n=174) and Met/Met individuals (n=17) (Figure **1**). We did not find a significant interaction between rs6265 and age on any of the cognitive phenotypes. We also did not find a significant interaction between Val66Met and sex in our sample with any of the cognitive or imaging phenotypes.

## Discussion

Our imaging-genetics analysis in a large dataset suggests that *BDNF* genetic variation may play a role in AD-related cognitive deficits as well as brain neurodegeneration. While we did not find an association between any of the *BDNF* SNPs and AD diagnosis, most likely due to low power, our analysis of *BDNF* SNPs in this large dataset confirms and extends a growing number of studies showing a relationship between *BDNF* genetic variation and both memory-related cognitive performance and brain morphometry in aging individuals, independent of APOE.

We found that there was a significant relationship between the *BDNF* Val66Met (rs6265) SNP and percent of right hippocampal atrophy over two years in nondemented individuals. Moreover we found that individuals who were heterozygous for the minor-allele of rs10501087, which is in LD with rs6265, had greater atrophy in the right hippocampal cortex than those who were homozygous for the major allele. Val66Met is a common functional polymorphism in the pro-region of *BDNF* known to mediate intracellular trafficking of proBDNF, and the Met-allele has been associated with disrupted cellular processing and secretion of BDNF [13]. In mammals, BDNF is highly expressed in the hippocampus [28]. In our analysis of rs6265, the Met-carrier group (minor-allele carrying group) was associated with higher rates of atrophy in the hippocampus over time, an AD-related marker of neurodegeneration. A recent study of glucose metabolism (FDG-PET) in the ADNI dataset also found that Met carriers compared with noncarriers had AD-like glucose metabolism in memory-related regions such as the temporal, parietal, occipital and hippocampal cortices [24]. Studies have also shown that the *BDNF* Val66Met Met-allele carriers have impaired episodic memory, decreased hippocampal volume, as well as reduced hippocampal activity during declarative memory processing [12-14,29,30]. Moreover, a recent study in the Australian Imaging, Biomarkers and Lifestyle study found that healthy adults with high Aβ levels who also had a Met allele of *BDNF* rs6265 had significant declines in episodic memory, executive function, and greater hippocampal atrophy over 3 years [31]. This, along with our data, argues that the Met-allele may contribute to brain change associated with preclinical AD in aging individuals. However, a recent study showed an interaction between *BDNF* Val66Met and age such that with increasing age, it was the Val/Val individuals that had decreased cortical thickness measures, decreased performance on episodic memory tasks, and reductions in white matter fractional anisotropy [16]. In addition, one of the largest studies on this SNP recently reported reductions in white matter fractional anisotropy (also a marker of neurodegeneration) in Val/Val homozygotes of the Val66Met SNP in prefrontal and occipital pathways, as well as correlations between cognitive performance and loss of white matter tract integrity [32]. This study was recently corroborated by a second analysis of diffusion tensor measures in healthy individuals in which they found that the Val allele was associated with abnormal white matter microstructure [33]. Although our analysis in ND individuals revealed that Met-allele carriers had significantly increased hippocampal atrophy, we found a significant interaction between age and baseline whole brain volume and *BDNF* rs6265 SNP in our combined diagnosis group, which showed that Val/Val individuals have lower whole brain volumes with increasing age compared to the Met/Met homozygotes and Met-allele carriers. It could also be that the Met and Val-allele variations function differently in individuals with ND verses those with AD, as another recent study on Val66Met in MCI and AD patients found that executive function was decreased in Val/Val homozygotes with AD compared to Met carriers [34].

A number of studies have investigated other SNPs in the *BDNF* gene and their association with various diseases as well as phenotypic markers of disease. For instance, a study of candidate genes for AD in a large French sample found that rs6265 was not associated with AD risk (nor was rs1157659, rs11030108, rs908867, rs1491850), however SNP rs11030094 was significantly associated with AD (p=.01, odds ratio .91), even when adjusted for other AD risk genes such as *APOE*, *CLU, CR1*, and *PICALM* [35]. In our sample we found that rs11030094 was associated with cognitive decline over 1 year in the nondemented group, baseline hippocampal volume (a trend at p=.056) and whole brain atrophy in the nondemented group. Similarly, other tagging SNPs implicated in risk for depression as well as anti-depressant treatment response, such as rs1491850, rs10501087, rs908867 [36,37], were both related to increased hippocampal atrophy in our sample of elderly individuals, highlighting the complexity of this gene and its involvement in brain disease. Another study found an association between a SNP in the *BDNF* gene (C270T), a SNP that has been associated with late-onset AD in some but not all studies [19,38-40], and executive function in patients with Alzheimer’s disease [41]. Circulating serum levels of *BDNF* decrease with age, and serum BDNF may mediate age-related hippocampal decline [42]. Moreover, postmortem research has shown reductions in hippocampal *BDNF* levels in elderly individuals, as well as even lower levels in individuals with AD [43,44]. While we did not measure either circulating or brain levels of *BDNF*, our results build on these previous studies associating *BDNF* genotype, which may impact *BDNF* protein levels, and hippocampal deterioration. Studies using brain imaging phenotypes of aging and AD as outcomes for tests of the role of genetic variation may be more sensitive to the actual impact of the gene on heterogeneous, complex diseases than typical diagnostic outcomes, as they are closer to the effect of the gene.

We tested whether several SNPs were implicated in AD-related cognitive decline, as measured by change in the ADAS-Cog test over one and two years; while we did not find a relationship between the Val66Met SNP and cognitive outcomes, we found several SNPs associated with this measure of progression (rs1157659, rs11030094, rs11030108) independent of ApoE4. Another study also found no relationship between Val66Met (and C270T and G712-A) and rates of cognitive decline in AD, however they did not include our additional SNPs in their analysis [45]. In fact, to our knowledge, there have been no reports on many of our tested SNPs, for instance rs1157659, which was associated with both ADAS scores and hippocampal atrophy in our sample. Thus, it may be that some SNPs in *BDNF*, but not others, impact the clinical course of AD, and those studies testing only a few of the SNPs in the gene may miss relationships that otherwise are associated with *BDNF*.

One limitation to this study could be reduced sensitivity of our chosen AD phenotypes to actual AD-related neurodegeneration, as we limited our analyses to test hippocampal and whole brain volume, and recent studies have begun using extended brain regions such as the temporal pole, inferior lateral ventrical, and precuneus as brain imaging markers of risk. We did not find a relationship between *BDNF* SNPs and cognitive measures in the AD group. This could be because the AD group has less variance on these measures, or perhaps because *BDNF* primarily influences aging-related cognitive decline, as seen in the AD group, but not dramatic disease-related decline as seen in the AD group. Furthermore, brain imaging measures of cognitive task related functional change (functional-MRI) would be more useful in characterizing the relationship between *BDNF* and hippocampal functionality during memory-related tasks. This data may become available in an ADNI subset for future investigations. In addition, we did not find a relationship between *BDNF* SNPs and imaging phenotypes in the MCI group. The other study using PET measures to study *BDNF* in individuals with MCI did find significant relationships, however they used a voxel-based approach, which may be useful for future studies interested in regions other than the medial temporal cortex. Moreover, the ADNI sample is limited due to a specific age range included in the study and interactions of age and genetic variation on brain volume will be more informative when including a larger range of ages. The ADNI sample is also limited in sample size when considering longitudinal imaging data of specific genotype and diagnosis subgroups. While we used Bonferroni-corrected p-values due to the number of tests across various phenotypes and genotypes, it will still be important to replicate these findings in a second data set, and ideally in a larger dataset. Because of possible functionality of several SNPs identified in our study and others (ex. rs908867, rs149850, rs10501087), it will be important to identify the genetic mechanisms of these SNPs in future studies. Finally, our sample was limited to Caucasians to avoid genetic stratification across ethnicities, however *BDNF* may have differing frequencies and polymorphisms across ethnicities that would not be represented in our results. Overall, we found that while BDNF genetic variation is not specifically associated with AD, it may play a significant role in aging or AD-related brain neurodegeneration, specifically in the hippocampus.

## Supporting Information

Table S1 **Linkage Disequilibrium map for *BDNF* SNPs.** Values in gray represent r^2^ and values in blue represent d-prime.
(DOCX)
