# Supplementary figures and images for: Characterizing the Role of Brain Derived Neurotrophic Factor Genetic Variation in Alzheimer’s Disease Neurodegeneration
Source: PLoS One. 2013 Sep 26;8(9):e76001. doi: 10.1371/journal.pone.0076001 (PMC3784423; doi:10.1371/journal.pone.0076001)

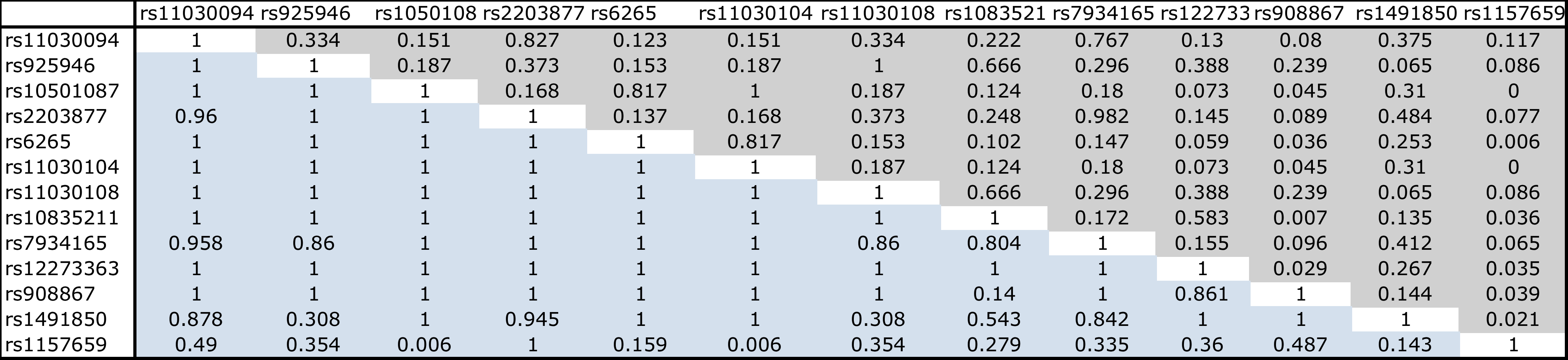

Supplement: Table S1 — Linkage Disequilibrium map for BDNF SNPs. Values in gray represent r2 and values in blue represent d-prime. (DOCX) [file pone.0076001.s001.docx]
